# Activated Drp1 regulates p62-mediated autophagic flux and aggravates inflammation in cerebral ischemia-reperfusion via the ROS-RIP1/RIP3-exosome axis

**DOI:** 10.1186/s40779-022-00383-2

**Published:** 2022-05-27

**Authors:** Xue Zeng, Yun-Dong Zhang, Rui-Yan Ma, Yuan-Jing Chen, Xin-Ming Xiang, Dong-Yao Hou, Xue-Han Li, He Huang, Tao Li, Chen-Yang Duan

**Affiliations:** 1 Department of Anaesthesiology, the Second Affiliated Hospital of Chongqing Medical University, 400010 Chongqing, China; 2 Department of Neurology, the Third Affiliated Hospital of Chongqing Medical University, Chongqing, 401120 China; 3 Department of Cardiovascular Surgery, Xinqiao Hospital, Army Medical University, Chongqing, 400037 China; 4 State Key Laboratory of Trauma, Burns and Combined Injury, Department of Shock and Transfusion, Daping Hospital, Army Medical University, Chongqing, 400042 China

**Keywords:** Cerebral ischemia-reperfusion (CIRI), Oxygen–glucose deprivation/reoxygenation (OGD/R), Drp1, p62, LC3 II/I, Reactive oxygen species (ROS), RIP1/RIP3, Autophagy, Exosome, Inflammatory

## Abstract

**Background:**

Cerebral ischemia-reperfusion injury (CIRI) refers to a secondary brain injury that can occur when the blood supply to the ischemic brain tissue is restored. However, the mechanism underlying such injury remains elusive.

**Methods:**

The 150 male C57 mice underwent middle cerebral artery occlusion (MCAO) for 1 h and reperfusion for 24 h, Among them, 50 MCAO mice were further treated with Mitochondrial division inhibitor 1 (Mdivi-1) and 50 MCAO mice were further treated with N-acetylcysteine (NAC). SH-SY5Y cells were cultured in a low-glucose culture medium for 4 h under hypoxic conditions and then transferred to normal conditions for 12 h. Then, cerebral blood flow, mitochondrial structure, mitochondrial DNA (mtDNA) copy number, intracellular and mitochondrial reactive oxygen species (ROS), autophagic flux, aggresome and exosome expression profiles, cardiac tissue structure, mitochondrial length and cristae density, mtDNA and ROS content, as well as the expression of Drp1-Ser616/Drp1, RIP1/RIP3, LC3 II/LC3 I, TNF-α, IL-1β, etc., were detected under normal or Drp1 interference conditions.

**Results:**

The mtDNA content, ROS levels, and Drp1-Ser616/Drp1 were elevated by 2.2, 1.7 and 2.7 times after CIRI (*P* < 0.05). However, the high cytoplasmic LC3 II/I ratio and increased aggregation of p62 could be reversed by 44% and 88% by Drp1 short hairpin RNA (shRNA) (*P* < 0.05). The low fluorescence intensity of autophagic flux and the increased phosphorylation of RIP3 induced by CIRI could be attenuated by ROS scavenger, NAC (*P* < 0.05). RIP1/RIP3 inhibitor Necrostatin-1 (Nec-1) restored 75% to a low LC3 II/LC3 I ratio and enhanced 2 times to a high RFP-LC3 after Drp1 activation (*P* < 0.05). In addition, although CIRI-induced ROS production caused no considerable accumulation of autophagosomes (*P* > 0.05), it increased the packaging and extracellular secretion of exosomes containing p62 by 4 – 5 times, which could be decreased by Mdivi-1, Drp1 shRNA, and Nec-1 (*P* < 0.05). Furthermore, TNF-α and IL-1β increased in CIRI-derived exosomes could increase RIP3 phosphorylation in normal or oxygen–glucose deprivation/reoxygenation (OGD/R) conditions (*P* < 0.05).

**Conclusions:**

CIRI activated Drp1 and accelerated the p62-mediated formation of autophagosomes while inhibiting the transition of autophagosomes to autolysosomes via the RIP1/RIP3 pathway activation. Undegraded autophagosomes were secreted extracellularly in the form of exosomes, leading to inflammatory cascades that further damaged mitochondria, resulting in excessive ROS generation and the blockage of autophagosome degradation, triggering a vicious cycle.

**Supplementary Information:**

The online version contains supplementary material available at 10.1186/s40779-022-00383-2.

## Background

The brain is particularly sensitive to ischemia and hypoxia, and ischemic cerebrovascular disease is the second leading cause of mortality among human diseases [1]. Mechanical thrombectomy and the intravenous infusion of the plasminogen activator are often used to restore the blood supply to ischemic brain tissue as soon as possible when treating ischemic cerebrovascular disease; however, the secondary damage that affects brain function following reperfusion, clinically known as cerebral ischemia-reperfusion injury (CIRI), remains difficult to address [2]. CIRI is a complex pathophysiological process involving energy disorders, cellular acidosis, the increased release of excitatory amino acids, the loss of intracellular calcium homeostasis, free radical generation, and the activation of apoptotic genes, which are mutually causal and intertwined, forming a vicious cycle that ultimately leads to apoptosis or necrosis [3]. Mitochondrial dysfunction is closely related to CIRI and is a key target area involved in the death of nerve cells following ischemia. Currently, the mechanisms triggering the vicious cycle of CIRI are still under exploration [4]. Investigating the mechanism by which CIRI causes damage via mitochondrial injury may be key to interrupting the vicious cycle, yielding effective intervention targets for CIRI.

Mitochondria maintain the dynamic balance of cells through continuous fission, fusion, autophagy, generation, and other processes that are key to maintaining normal mitochondrial function [5]. Acute ischemic injuries, such as sepsis, shock, or pulmonary hypertension, affect the mitochondrial mass balance causing excessive mitochondrial fission and abnormal mitophagy [6]. Haileselassie et al. [7] showed that excessive mitochondrial fission could lead to increased vascular permeability, disruption of the blood–brain barrier, and infectious encephalopathy in a murine model of sepsis. Our previous study [8] in a mouse model of hemorrhagic shock showed that excessive mitochondrial fission and abnormal autophagy could lead to mitochondrial dysfunction, resulting in reduced ATP synthesis, massive reactive oxygen species (ROS) accumulation, and reduced mitochondrial membrane potential, ultimately leading to cardiovascular dysfunction [9, 10] and the disruption of the intestinal barrier function [11]. We also found that changes in the expression and activity of the mitochondrial fission-related protein Drp1 and autophagy-related protein p62 play an important role in ischemia-hypoxia-induced oxidative stress injury.

As a key protein regulating mitochondrial fission, Drp1 is usually freely distributed within the cytoplasm; however, its activation following hypoxic injury leads to its translocation from the cytoplasm to the mitochondrial surface, where it cleaves the mitochondrial phospholipid bilayer via GTPase, initiating mitochondrial fission and fragmentation [10]. In myocardial ischemia-reperfusion, Drp1-Ser616 phosphorylation and Drp1 translocation to the mitochondria can lead to massive ROS accumulation and accelerate cardiomyocyte necrosis [12]. In hepatic ischemia-reperfusion, the dephosphorylation of Drp1-Ser637 results in calcium overload in the mitochondrial matrix, inducing hepatic encephalopathy [13]. As a selective autophagy adaptor protein, p62 is the primary defense protein regulating the autophagic degradation of mitochondrial fragments following oxidative stress. p62 binds to LC3 on the mitochondrial membrane to form autophagosomes, which are degraded to autolysosomes, triggering autophagic flux and accelerating the clearance of protein aggregates [14]. In acute myocardial infarction, the p62-mediated disturbance of autophagic flux could accelerate cardiomyocyte lysis and lead to autophagic cell death [15]. However, whether the two key processes underlying mitochondrial quality imbalance, Drp1-mediated mitochondrial fission and p62-mediated autophagic flux, are altered by CIRI injury, whether an internal correlation exists between these two processes, and how Drp1 and p62 are involved in regulating the vicious cycle of CIRI remains unclear. To date, no relevant report has attempted to answer these questions.

In this study, middle cerebral artery occlusion (MCAO) model mice and an in vitro model of oxygen–glucose deprivation/reoxygenation (OGD/R) in SH-SY5Y cells were used to clarify the occurrence of Drp1- and p62-mediated mitochondrial quality imbalance after CIRI. Then, mitochondrial quality detection (such as observation of mitochondrial morphology, content, ROS measurement, etc.) and autophagic flux assay were performed to explore the regulatory mechanisms of Drp1 activation on p62-mediated autophagosome formation as well as degradation following CIRI. We finally elaborated on the critical role of mitochondrial quality imbalance in the vicious cycle underlying CIRI. It is suggested that the trigger of CIRI might be the mitochondrial quality imbalance mediated by the activated Drp1, and the key factor responsible for the vicious cycle underlying CIRI may be the massive ROS accumulation. The deterioration associated with CIRI is thought to result from blockage of the positive feedback autophagic flow that occurs in the RIP1/RIP3 pathway and the release of massive amounts of exosomes containing p62. Our study systematically investigated the pathophysiological process of CIRI vicious cycle from the perspective of mitochondrial damage, providing an experimental basis and intervention target for the prevention and treatment of CIRI.

## Methods

### Materials

Antibodies against Drp1, p62, LC3, RIP1, RIP3, TSG101, and CD63 were purchased from Abcam (Cambridge, MA, USA). β-actin, tubulin, and COX IV, which were used as references for the total, cytoplasmic, and mitochondrial fractions, were also acquired from Abcam (Cambridge, MA, USA). Antibodies against Phospho-RIP3 (Thr231/Ser232) and Phospho-Drp1 (Ser616) were purchased from Cell Signaling Technology (Danvers, MA, USA). The Aggresome Detection Kit was acquired from Abcam (Cambridge, MA, USA). MitoTracker Deep Red, the ROS 2′,7′-dichlorodihydrofluorescein diacetate (DCFH-DA) kit, dihydroethidium (DHE) assay kit, and MitoSOX detection kit were purchased from Invitrogen (Carlsbad, CA, USA). Ad-mRFP-GFP-LC3 was supplied by Beyotime (Shanghai, China). Adenoviral vectors for Drp1 short hairpin RNA (shRNA) and Drp1 mutations (Drp1-S616D and Drp1-S616A) were generated by Genechem Technology (Shanghai, China). Related activators and inhibitors, including Mitochondrial division inhibitor 1 (Mdivi-1), N-acetylcysteine (NAC), and Necrostatin-1 (Nec-1), were obtained from Selleck (Shanghai, China). The Protein A/G Magnetic Beads IP Kit was purchased from Thermo Scientific (Waltham, MA, USA), and fetal bovine serum (FBS) and penicillin/streptomycin were procured by Invitrogen (Carlsbad, CA, USA). All other chemicals were supplied by Sigma unless otherwise specified.

### Model preparation

#### MCAO

The 200 male C57 mice (8-week-old, 21–25 g) were fasted overnight but allowed free access to water (Normal group: 50 mice, MCAO group: 50 mice, MCAO + Mdivi-1 group: 50 mice, MCAO + NAC group: 50 mice). As for the procedure of MCAO model, anesthesia was induced with 3.5% halothane in a mixture of 70% nitrous oxide balanced with oxygen. Under an operating microscope, the left common carotid artery was exposed through a midline neck incision and carefully dissected from the surrounding nerves and fascia from its bifurcation to the skull base. The occipital artery branches of the external carotid artery were then isolated, dissected, and coagulated. The internal carotid artery was isolated and carefully separated from the adjacent vagus nerve, and the pterygopalatine artery was ligated. Next, a threaded plug with a 0.20-mm-diameter silicone head (RWD Life Science, Shenzhen, China) was inserted into the internal carotid artery via the proximal external carotid artery and then into the circle of Willis, effectively occluding the middle cerebral artery. After the left middle cerebral artery was blocked for 1 h, the threaded plug was pulled out to allow blood to reperfuse into the internal carotid artery for 24 h. All procedures were approved by the Laboratory Animal Welfare and Ethics Committee of Army Medical University (AMUWEC20171288). The investigation conformed to the Guide for the Care and Use of Laboratory Animals (National Institutes of Health, Publication No. 85–23, Revised 1996).

#### Cell OGD/R treatment

SH-SY5Y cells were cultured in low-glucose Dulbecco’s modified Eagle medium (DMEM, 1000 mg/L d-glucose) without FBS, transferred into a hypoxic culture compartment (MIC-101, Billups-Rothenberg, Del Mar, CA, USA), with 95% N_2_ and 5% CO_2_ with an estimated oxygen concentration of < 0.2%, and incubated for 4 h under hypoxic conditions as described previously [10]. The culture medium was then changed to DMEM-F12 (Gibco, NY, USA) supplemented with 10% FBS (Hyclone, Logan, UT, USA), and cells were incubated at 37 ℃ for 12 h.

### Speckle tomography of cerebral blood flow

Mice were laid prostrate on the board after anesthesia, and the scalp was fully exposed. A low-energy He–Ne laser probe was placed 14 cm above the mouse skull, and the blood flow was observed using a laser Doppler perfusion imager (PeriCam PSI ZR, Sweden) as described previously [10]. Blood flow was calculated using blood flow analysis software PIMsoft (Version 1.5, Perimed AB, Järfälla, Sweden).

### Mitochondrial morphology

Mitochondria in SH-SY5Y cells were labeled using MitoTracker Deep Red (100 nmol/L) at 37 ℃ for 30 min and observed by confocal microscopy (Leica TCS SP5, Wetzlar, Germany) with a 60 × 1.3 NA oil-immersion objective. Mitochondrial fluorophores were excited with a 633 nm laser, and fluorescence emissions were recorded at 558–617 nm. The length of mitochondria was determined and calculated using Image J software.

### Mitochondrial DNA (mtDNA) copy number detection

The mtDNA copy number detection was performed to determine the number of mitochondria. Total DNA was extracted from cerebral cortex tissue. The amount of mitochondrial DNA relative to nuclear DNA was determined by quantitative real-time PCR using primers for Nd2 (mitochondrial genome, Rn03296765_s1; Invitrogen) and GAPDH (nuclear genome, Rn01775763_g1; Invitrogen). The relative mtDNA copy number was calculated based on a threshold cycle (Ct) of 2^−Δ(ΔCt)^, where ΔCt = Ct_Nd2 _− Ct_GAPDH_ and Δ(ΔCt) = ΔCt _sample _− ΔCt _control_ [10].

### Measurement of intracellular ROS and mitochondrial ROS

The measurement of intracellular ROS, including DCFH-DA detection and DHE in SH-SY5Y cells, was performed according to the kit instructions, as previously described [10]. Briefly, cells were incubated with 10 μmol/L DCFH-DA or DHE for 30 min at 37 ℃. DCFH-DA or DHE fluorescence was detected at 488 nm excitation and 501–563 nm emission by confocal microscopy (Leica TCS SP5, Wetzlar, Germany). The mean intensities of the DCFH-DA fluorescence were calculated using Leica TCS software. The DHE fluorescence intensity (arbitrary units, A.U.) of the SH-SY5Y cells was analyzed in a blinded fashion using ImageJ software.

Mitochondrial ROS were measured based on the fluorescence results of MitoSOX live-cell imaging (5 μmol/L for 10 min, 37 ℃). The MitoSOX red fluorescence of SH-SY5Y cells was detected at 580 nm excitation and 510 nm emission by confocal microscopy (Leica TCS SP5, Wetzlar, Germany). The mean intensities of MitoSOX red fluorescence were calculated using ImageJ software.

### Autophagic flux assay

SH-SY5Y cells were transfected with Ad-mRFP-GFP-LC3 (Beyotime Biotechnology, Shanghai, China) for 24 h to detect autophagy flux based on our previous study [8]. Strong red fluorescent LC3 proteins were assumed to indicate that autophagosomes had been engulfed in the lysosomal acidic environment to form autolysosomes, indicating a smooth mitophagy flux. In contrast, strong green fluorescent LC3 proteins indicated that autophagosomes could not be converted to autolysosomes, suggesting that autophagy was blocked.

### Aggresome detection

Aggresome detection was carried out according to the kit instructions [16]. Briefly, SH-SY5Y cells were plated in a confocal chamber and treated with an experimental test agent. The positive control was prepared by incubating cells with the diluted proteasome inhibitor MG-132. After fixing with 4% formaldehyde for 30 min, cells were permeabilized with 0.5% Triton-X 100 for 2 min, washed with PBS, blocked with 5% BSA at 37 ℃ for 1 h, and incubated with aggresome detection reagent at 37 ℃ for 1 h in the dark. Cells were then rewashed with PBS for 5 min and stained with DAPI (BD Biosciences, Franklin Lakes, NJ, USA) (1:50) before being visualized under a confocal laser-scanning microscope (Leica SP5, Germany).

### Public GEO data analysis

The data obtained from the in vivo model, GEO (GSE23160), and a proteome dataset from Wen et al. [17], were examined and visualized using R/Bioconductor. Individual gene expression was defined as altered when comparing the average normalized signal intensities with the Bioconductor package Genefilter gave a value of *P* < 0.05 in Welch’s analysis of variance (ANOVA). To investigate the ontology of differentially expressed genes, the enriched expression of GO terms was assessed and confirmed with the hypergeometric test using the Bioconductor package GoStats. This program determines which GO terms identified among the lists of affected genes are statistically over- or underrepresented compared to the GO terms represented in the microarray as a whole. The result from the hypergeometric test of a given GO in a pairwise comparison group is a pair of *P* values, which increase if overrepresented and decrease if underrepresented. The *P* values were visualized in the heatmap (right: increase; left: decrease, in each paired comparison). Hierarchical clustering was performed using Ward’s method to calculate the linkage distances based on the correlation coefficient between GO terms.

### Exosome extraction

Exosome extraction was achieved by the ultracentrifugation method based on a previous study [18]. Tissue or cell samples were centrifuged for 20 min at 10,000 × g and 4 ℃, and then the supernatant was filtered through a 0.22 um filter membrane. The filtrate was then centrifuged at 120,000 × g at 4 ℃ for 2 h. The supernatant was then carefully removed, and the precipitate was washed with an equal volume of cold PBS and centrifuged at 120,000 × g at 4 ℃ for 2 h. The precipitate was resuspended in 200 µL cold PBS and stored at –80 ℃ for nanoparticle tracking analysis (NTA), transmission electronic microscopy (TEM) observation, and protein detection. NTA detection was conducted with the assistance of Genechem Technology (Shanghai, China). The size and concentration of exosomes were analyzed using ZetaView SP2 software (Particle Metrix, Germany). Equal volumes of exosome supernatant were loaded into each well for immunoblotting, and the number of exosomes was quantified using CD63 and TSG101 as markers.

### Co-immunoprecipitation (Co-IP)

Co-IP was performed using the Protein A/G Magnetic Beads IP Kit according to the manufacturer’s instructions. The targeted antibody (10 mg) was diluted with 200 μl PBST. Protein A/G magnetic beads (50 μL) were added to the mixture, which was then incubated for 10 min at room temperature, and the antibody-conjugated immunomagnetic beads were prepared after the supernatant was removed from the magnetic separator. After the samples were harvested, lysed, and centrifuged, the supernatants were gently mixed with antibody-conjugated immunomagnetic beads to prepare an immunomagnetic beads-antibody-antigen complex. After washing the beads with PBS three times, the complex was resuspended in 100 μl PBS and used to detect endogenous interactions between the targeted antibody and other proteins by western blotting.

### HE staining

The cerebral cortex tissue was fixed for at least 2 h in 10% neutral buffered formalin; sections were then frozen for 3–5 min and mounted. After thermal antigen repair, sample slides were immersed in hematoxylin for 3 min and eosin dye for 1 min. Stained samples were then treated with gradient alcohol and xylene and sealed with gum for microscopic observation.

### Immunohistochemistry staining

Cerebral cortex tissue samples were cut into 5-mm-thick sections and placed onto silane-coated glass slides. Sections were deparaffinized, processed for antigen retrieval and blocking, and incubated overnight with primary antibodies against Drp1, P62, and CD63, followed by incubation with secondary antibodies on a peroxidase-labeled dextran polymer. Color development for immunohistochemistry was achieved using 3,3′-diaminobenzidine in 50 mmol/L Tris–HCl (pH 5.5) containing 0.005% hydrogen peroxidase. Finally, sections were counterstained with hematoxylin, and DAPI was pipetted onto each slide and left for 5 min at room temperature. Slides were then covered with ProLong^®^ Diamond Antifade Mountant (Thermo Scientific, USA), and images were acquired under a fluorescence microscope (IX-73, Olympus, Japan).

### Immunofluorescence staining

Immunofluorescence staining was performed on both cerebral cortex tissue samples and SH-SY5Y cells as previously described [10]. Sample slides were fixed in a 4% paraformaldehyde solution for 20 min, permeabilized with 0.1% Triton-X 100 in 1X PBS for 5 min, and blocked in a 5% BSA solution for 1 h at 37 ℃, after which they were washed and incubated with primary antibodies against Drp1, COX IV, p62, LC3, or CD63 overnight at 4 ℃. Samples were then washed in PBS with 0.1% Tween-20 and incubated with the corresponding fluorophore-conjugated mouse or rabbit secondary antibodies (Invitrogen, Carlsbad, CA, USA) for 1 h at room temperature before being subjected to final washing in 1 × PBS and incubated with DAPI (BD Biosciences, Franklin Lakes, NJ, USA) (1:50) for 5 min at room temperature. Immunofluorescence was visualized under a confocal laser-scanning microscope (Leica SP5, Germany).

### TEM imaging

Fresh cerebral cortex tissue samples were obtained and fixed as described previously [8]. Samples were imaged using an H-7500 transmission electron microscope (Hitachi, Japan). The mitochondrial length and cristae density in the cerebral tissue were calculated using Image J software.

For exosome detection, 20 µL of exosome suspension was dropped onto a fixed carbon net and allowed to stand at room temperature for 20 min, after which the suspension containing the excess secretion was carefully removed. Twenty microliters of 2% phosphotungstic acid were then dropped onto the carbon net, which was left to stand for 20 s before being placed into a glass dish and covered with filter paper, and left until TEM observation was performed.

### Western blotting

Subcellular fractions, including the cytoplasmic and mitochondrial fractions, were isolated based on the kit instructions (Invent SC-003/NM-038; Beijing, China). β-actin, tubulin, and COX IV were used as internal references for total, cytoplasmic, and mitochondrial fractions, respectively. The fractioned and total proteins were then used for immunoblotting analyses with the indicated antibodies as previously described [10]. The intensity of the bands was analyzed using Quantity One V 4.62 software (Bio-Rad, Life Science, California, USA).

### Statistics analysis

Data were expressed as mean ± standard deviation. An independent sample *t*-test was used for the experiments with the two groups. One-way ANOVA was used for experiments with more than two groups, followed by Tukey’s post hoc analysis and SNK or LSD comparison. SPSS (version 17.0, IBM Corp., Armonk, NY, USA) was used with statistical significance set at *P* < 0.05.

## Results

### Activated Drp1 leads to excessive mitochondrial fission and ROS accumulation after CIRI

**Fig. 1:**
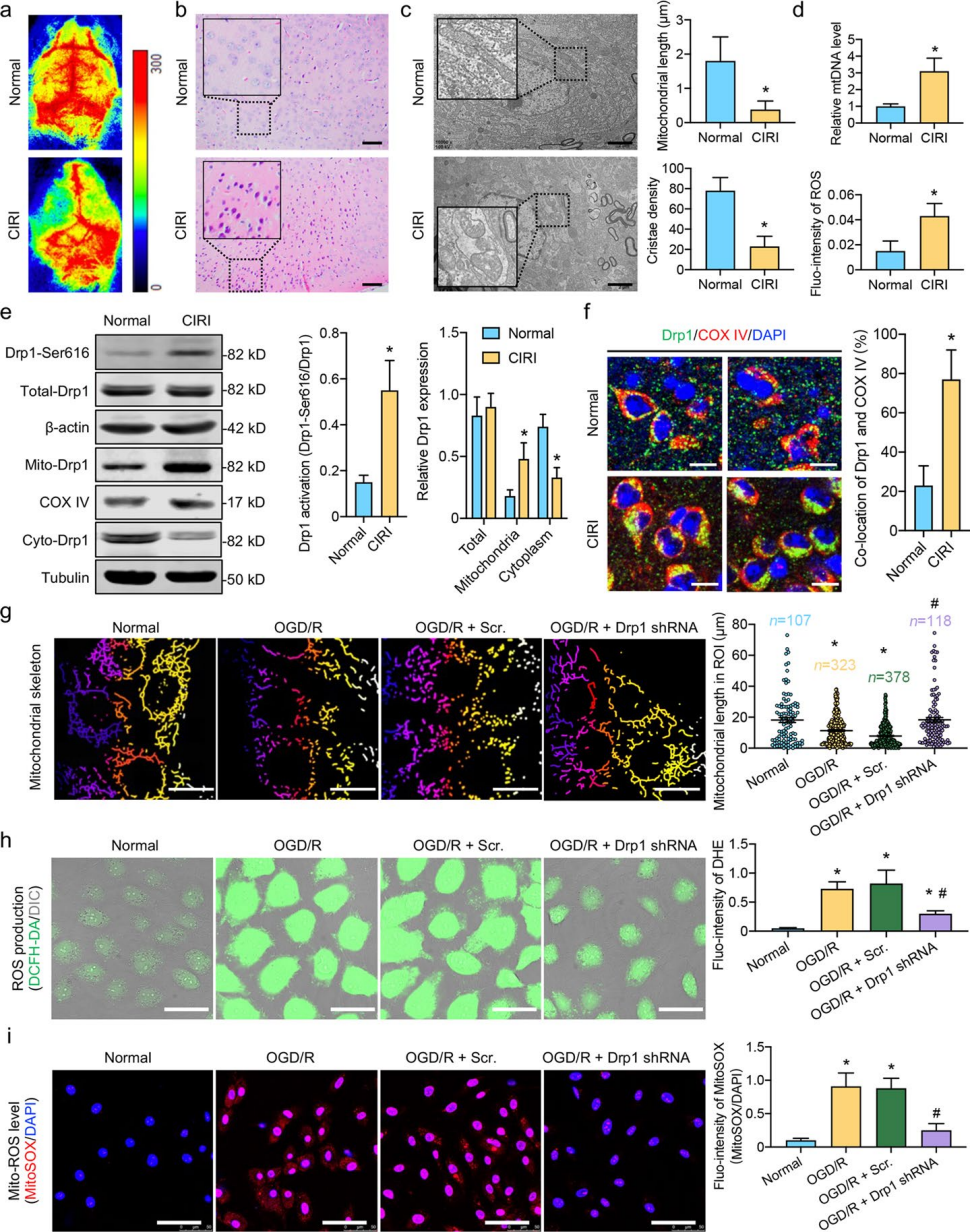
Excessive mitochondrial fission and ROS accumulation induced by activated Drp1 after CIRI and ODG/R. **a** Speckle tomography of cerebral blood flow after CIRI. **b** HE staining of the cerebral cortex after CIRI (bar = 50 μm). **c** TEM images showing mitochondria in the cerebral cortex after CIRI (bar = 2 μm). Quantitative data detailing the mitochondrial length and cristae density of each group. **d** mtDNA copy number and fluorescence intensity of ROS DCFH-DA in the cerebral cortex after CIRI (*n* = 8/group). **e** Activation of Drp1-Ser616 as well as the protein expression of Drp1 in total, cytoplasmic, and mitochondrial fractions after CIRI. β-actin, COX IV, and tubulin were used as internal references for total, mitochondrial, and cytoplasmic fractions, respectively (*n* = 8/group). **f** Immunofluorescence images showing co-location of Drp1 and mitochondria (COX IV) in the cerebral cortex after CIRI. Enlarged images of Additional file 1: Fig. S1c (bar = 10 μm, *n* = 5/group). **g** Mitochondrial morphology in OGD/R-treated SH-SY5Y cells after Drp1 shRNA (bar = 25 μm). The length of mitochondria was analyzed using Image J software, with 107 mitochondria observed and measured in the normal group, 323 in the OGD/R group, 378 in the OGD/R + Scr. group, and 118 in the OGD/R + Drp1 shRNA group. **h** Fluorescence intensity of ROS DCFH-DA and quantitative data of DHE fluorescence intensity in OGD/R-treated SH-SY5Y cells after Drp1 shRNA (bar = 50 μm, *n* = 8/group). **i** Fluorescence images and related quantitative data detailing Mito-ROS MitoSOX fluorescence in OGD/R-treated SH-SY5Y cells after Drp1 shRNA (*n* = 8/group). ^*^P < 0.05, compared with normal group; ^#^*P* < 0.05, compared with OGD/R group. CIRI cerebral ischemia–reperfusion injury, DCFH-DA 2',7'-dichlorodihydrofluorescein diacetate, DHE dihydroethidium, Mito-ROS mitochondrial ROS, mtDNA mitochondrial DNA, OGD/R oxygen- glucose deprivation/reoxygenation treatment, ROI Region of interest, ROS reactive oxygen species, Scr. scramble-shRNA, TEM transmission electron microscope

The classical MCAO model was used to simulate the process of CIRI. Speckle tomography of cerebral blood flow showed a weak hemorheological signal on the side of the cerebral infarction (Fig. 1a). HE staining showed obvious inflammatory infiltration in the infarcted area of the cerebral cortex (Fig. 1b), indicating that the MCAO model was successfully established. The mitochondrial injury was apparent after CIRI, mainly reflected by excessive mitochondrial fission and abnormal mitochondrial function. TEM images revealed increased mitochondrial fragmentation and cristae vacuolation following CIRI. The mitochondrial length in the normal and CIRI groups was (1.8 ± 0.65) μm and (0.38 ± 0.19) μm, respectively; cristae densities in the normal and CIRI groups were (78 ± 12)% and (23 ± 9)%, respectively (*P* < 0.05, Fig. 1c). The mtDNA content and ROS levels in the infarcted area after CIRI increased 2.2 and 1.7 times, respectively, compared to those in the normal group (*P* < 0.05, Fig. 1d). Drp1, a mitochondrial fission-related protein, showed excessive aggregation, with the proportion of aggregated Drp1-positive cells in the IR group reaching 73%, which was significantly higher than that observed in the normal group (*P* < 0.05, Additional file 1: Fig. S1a). Western blotting indicated that the aggregated Drp1 was mainly characterized by mitochondrial translocation induced by the activation of Drp1-Ser616 following CIRI (Fig. 1e), which was also confirmed in vitro (Additional file 1: Fig. S1b). Immunofluorescence images showed that Drp1 and COX IV co-localization increased significantly (Fig. 1f, *P *< 0.05), further confirming that Drp1 accumulates on mitochondria following CIRI (Additional file 1: Fig. S1c). The same phenomena were also observed in SH-SY5Y cells undergoing OGD/R on a cellular level. Confocal images showed that the number of linear mitochondria decreased significantly after OGD/R, whereas the number of punctate mitochondria increased (*P* < 0.05), and this process could be reversed by Drp1 shRNA (Fig. 1g). The fluorescence intensity of both DCFH-DA and DHE increased (*P* < 0.05), indicating the accumulation of ROS (Fig. 1h), and the trends in the mitochondrial ROS (Mito-ROS) assayed using MitoSOX were consistent with the variations in the total ROS levels after ODG/R (Fig. 1i), further confirming that activated Drp1 may lead to mitochondrial ROS accumulation after OGD/R in vitro*.*

### Activated Drp1 accelerates the formation of p62-containing autophagosomes after CIRI

**Fig. 2:**
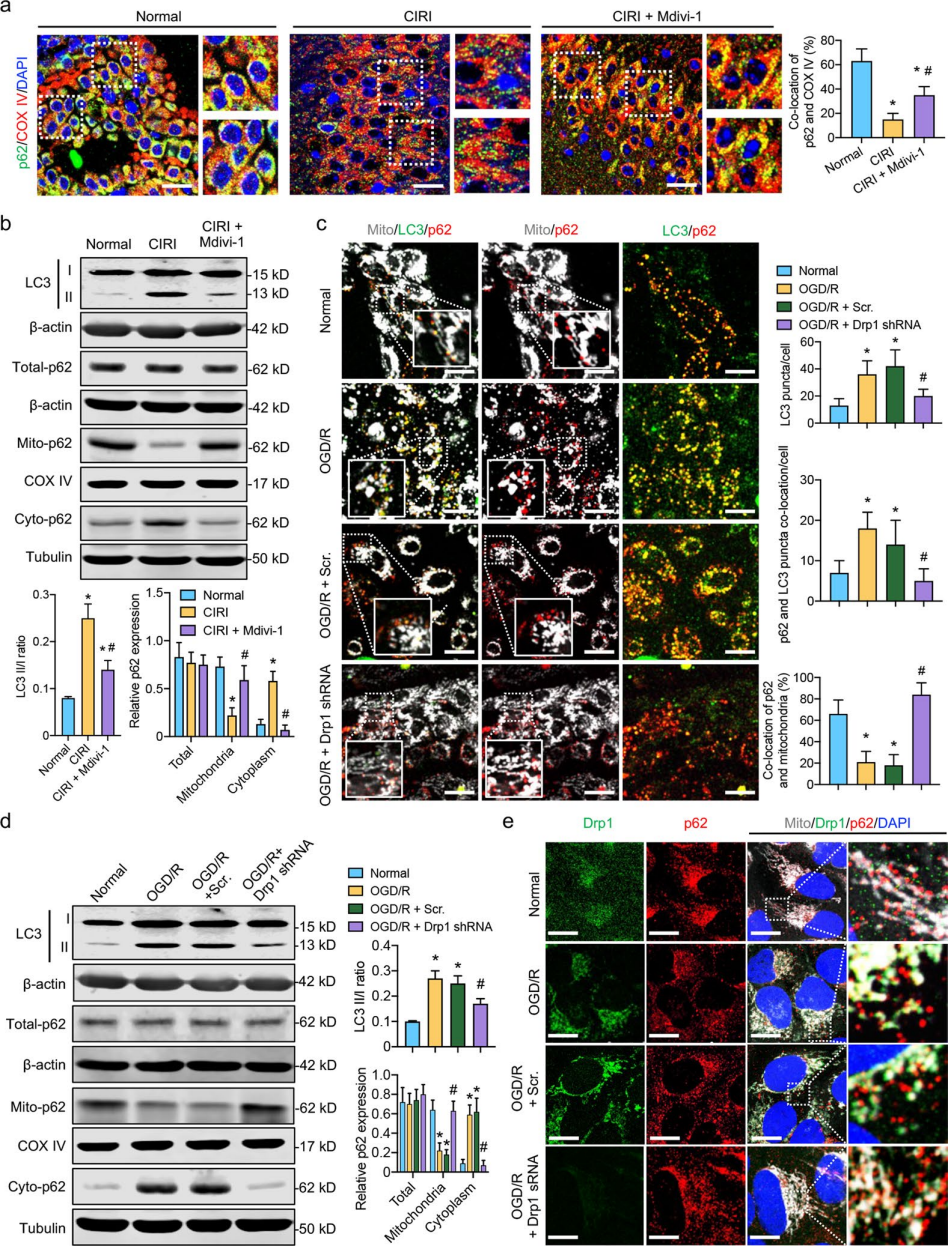
Effects of Drp1 intervention on p62-mediated autophagosome formation after CIRI and ODG/R. **a** Immunofluorescence images showing co-location of p62 and mitochondria (COX IV) in the cerebral cortex after CIRI and Mdivi-1 treatment (bar = 20 μm; *n* = 5/group). **b** LC3 II/I and the protein expression of p62 in total, cytoplasmic, and mitochondrial fractions after CIRI and Mdivi-1 treatment. β-actin, COX IV, and tubulin were used as internal references for total, mitochondrial, and cytoplasmic fractions, respectively (*n* = 8/group). **c** Confocal images showing co-location of LC3, p62, and mitochondria in OGD/R-treated SH-SY5Y cells after Drp1 shRNA (bar = 25 μm; *n* = 5/group). **d** LC3 II/I and protein expression of p62 in total, cytoplasmic, and mitochondrial fractions in OGD/R-treated SH-SY5Y cells after Drp1 shRNA. β-actin, COX IV, and tubulin were used as internal references for total, mitochondrial, and cytoplasmic fractions, respectively (*n* = 8/group). **e** Confocal images showing SH-SY5Y cells labeled with Drp1-GFP, p62-RFP, and MitoTracker after Drp1 shRNA (bar = 25 μm; *n* = 5/group). ^*^P < 0.05, compared with normal group; ^#^*P* < 0.05, compared with CIRI or OGD/R group. CIRI cerebral ischemia–reperfusion injury, OGD/R oxygen–glucose deprivation/reoxygenation treatment, Scr. scramble-shRNA

The immunohistochemistry images showed that the proportion of aggregated p62-positive cells in the cerebral infarction area decreased significantly after CIRI, whereas this proportion increased by 47% after the administration of Mdivi-1, a Drp1 inhibitor (*P* < 0.05, Additional file 1: Fig. S2a). The immunofluorescence images showed that the co-localization of p62 and COX IV-labeled mitochondria in the infarcted area decreased significantly after CIRI, whereas this co-localization increased by 40% after Mdivi-1 was administered (*P* < 0.05, Fig. 2a and Additional file 1: Fig. S2b). Western blotting analysis indicated that the LC II/I ratio increased significantly after CIRI but decreased by 36% after Mdivi-1 administration (*P* < 0.05, Fig. 2b). Further analysis showed that although the total p62 expression did not change significantly (*P* > 0.05), the translocation of p62 from mitochondria to cytoplasm increased markedly after CIRI and that this process could be reversed by inhibiting Drp1 activity with Mdivi-1 (Fig. 2b). These results suggest that CIRI-induced Drp1 activation may enhance the release of p62 from mitochondria and accelerate the formation of autophagosomes following CIRI.

Variations in the p62-containing LC3-autophagosomes were also observed in the OGD/R-treated SH-SY5Y cells. The confocal images showed that the number of LC3 puncta and p62-containing LC3-autophagosomes increased significantly after OGD/R. Further interference with Drp1 shRNA reduced the number of LC3 puncta and p62-containing LC3-autophagosomes by 52% and 64%, respectively (Fig. 2c), indicating that OGD/R-induced Drp1 activation accelerates the formation of p62-mediated autophagosomes in vitro. Western blotting analysis showed that the increased LC3 II/I ratio and p62 translocation induced by OGD/R could be reversed by Drp1 shRNA (Fig. 2d), consistent with the confocal microscopy results (Fig. 2c). We then transfected SH-SY5Y cells with Drp1-GFP and p62-RFP plasmids. The resulting immunofluorescence images showed that Drp1 was mostly diffused in the cytoplasm, and p62 was primarily aggregated in mitochondria under normal conditions. After OGD/R, a large amount of activated Drp1 was translocated to the mitochondria at the same time as mitochondrial p62 was translocated to the cytoplasm; however, the OGD/R-induced p62 release could be inhibited by Drp1 shRNA (Fig. 2e). These observations indicated that activated Drp1 might promote the release of mitochondrial p62 to the cytoplasm after OGD/R while not affecting p62 expression. Overall, the above in vivo and in vitro results confirmed that the mitochondrial translocation of activated Drp1 may accelerate the p62-mediated formation of autophagosomes following CIRI.

### Activated Drp1-induced ROS accumulation blocks autophagosome degradation via RIP1/RIP3 pathway after CIRI

**Fig. 3:**
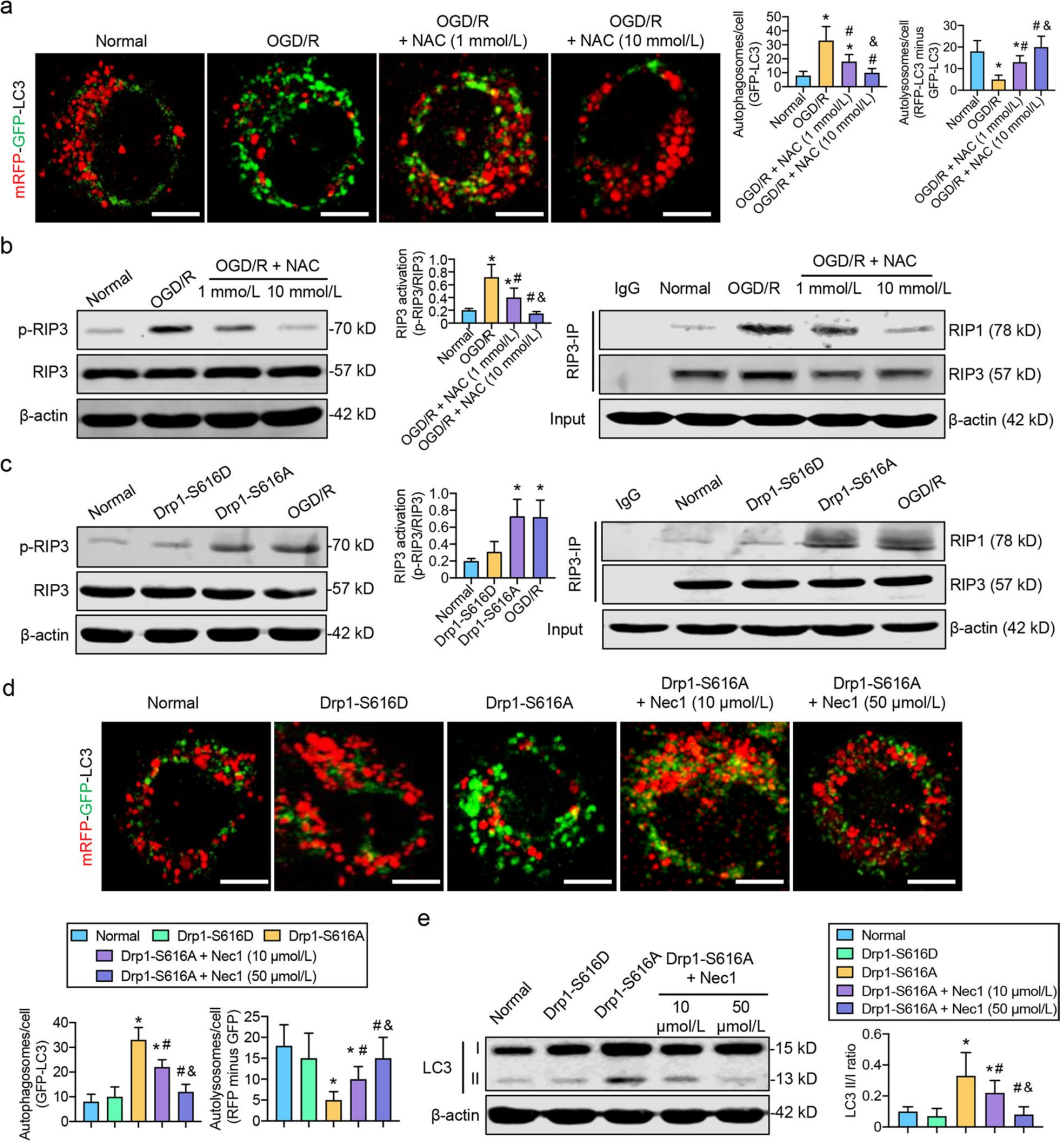
ROS accumulation blocks autophagosome degradation by activating the RIP1/RIP3 pathway after OGD/R. **a** Immunofluorescence images showing autophagy flux labeled by mRFP-GFP-LC3 in OGD/R-treated SH-SY5Y cells after NAC treatment (bar = 10 μm, *n* = 5/group). **b** Western blotting analysis and Co-IP assay showing RIP3 phosphorylation and RIP1-RIP3 binding ability in OGD/R-treated SH-SY5Y cells after NAC treatment (*n* = 8/group). **c** Western Blotting analysis and Co-IP assay showing RIP3 phosphorylation and RIP1-RIP3 binding ability in SH-SY5Y cells after Drp1 S616A mutation (*n* = 8/group). **d** Immunofluorescence images showing autophagy flux labeled by mRFP-GFP-LC3 in SH-SY5Y cells after Drp1 S616A mutation and Nec1 treatment (bar = 10 μm, *n* = 5/group). **e** Western Blotting analysis showing LC3 II/I ratio in SH-SY5Y cells after Drp1 S616A mutation and Nec1 treatment (*n* = 8/group). ^*^P < 0.05, compared with normal group; ^#^*P* < 0.05, compared with OGD/R or Drp1 S616A group; ^&^*P* < 0.05, compared with OGD/R + NAC (1 mmol/L) or OGD/R + Nec1 (10 μmol/L) group. Co-IP co-immunoprecipitation, OGD/R oxygen–glucose deprivation/reoxygenation treatment, ROS reactive oxygen species

Our previous results demonstrated that the CIRI-induced Drp1 activation might lead to excessive ROS accumulation and accelerate the formation of p62-containing autophagosomes. However, the regulatory relationship between these two Drp1-targeted pathways remains unclear. Therefore, we investigated the complete autophagic flux obtained by mRFP-GFP-LC3. In the normal group, autophagosomes could be phagocytized by lysosomes to form autolysosomes, and the high fluorescence intensity of “RFP-LC3 minus GFP-LC3” indicated a smooth autophagic flux. In OGD/R-treated SH-SY5Y cells, the excessive number of autophagosomes (GFP-LC3) could not be consumed by the lysosomal acidic environment, and the decrease in “RFP-LC3 minus GFP-LC3” fluorescence indicated that the p62-labeled autophagosomes could not fully transform into autolysosomes after OGD/R (Fig. 3a). The pretreatment of OGD/R-treated SH-SY5Y cells with the ROS scavenger, NAC, may significantly improve the transformation of autophagosomes to autolysosomes in a dose-dependent manner (Fig. 3a and Additional file 1: Fig. S3a), suggesting that p62-mediated autophagic flux may be related to Drp1-induced ROS accumulation post-OGD/R.

RIP1/RIP3 pathway plays an important role in the autophagic flux. We then detected RIP3 activity and its binding to RIP1 after OGD/R. The results indicated that RIP3 phosphorylation and the binding ability of RIP1 with RIP3 were significantly increased in OGD/R-treated SH-SY5Y cells. After ROS clearance with NAC, both RIP3 activity and RIP3/RIP1 binding were significantly decreased in a dose-dependent manner (Fig. 3b), which was also confirmed in NAC-treated CIRI mice (Additional file 1: Fig. S3b). These results indicate that OGD/R-induced ROS accumulation may block autophagic flux by activating the RIP1/RIP3 pathway.

Furthermore, we also used the Drp1-S616A mutation in SH-SY5Y cells to enhance Drp1 activity (Additional file 1: Fig. S3c). The results showed that RIP3 phosphorylation was significantly increased and the binding affinity of RIP1 and RIP3 was significantly enhanced (Fig. 3c) relative to the normal and Drp1-S616D groups (negative control). Further use of Nec-1, a RIP1/RIP3 inhibitor (Additional file 1: Fig. S3d, e), could significantly improve the transformation of autophagosomes to autolysosomes (Fig. 3d), promoting the p62-mediated degradation of autophagosomes (Fig. 3e). These results confirmed that activated Drp1-induced ROS accumulation might block autophagic flux via the RIP1/RIP3 pathway.

### Undegraded p62-labeled autophagosomes induced by activated Drp1 are secreted via exosomes after CIRI

**Fig. 4:**
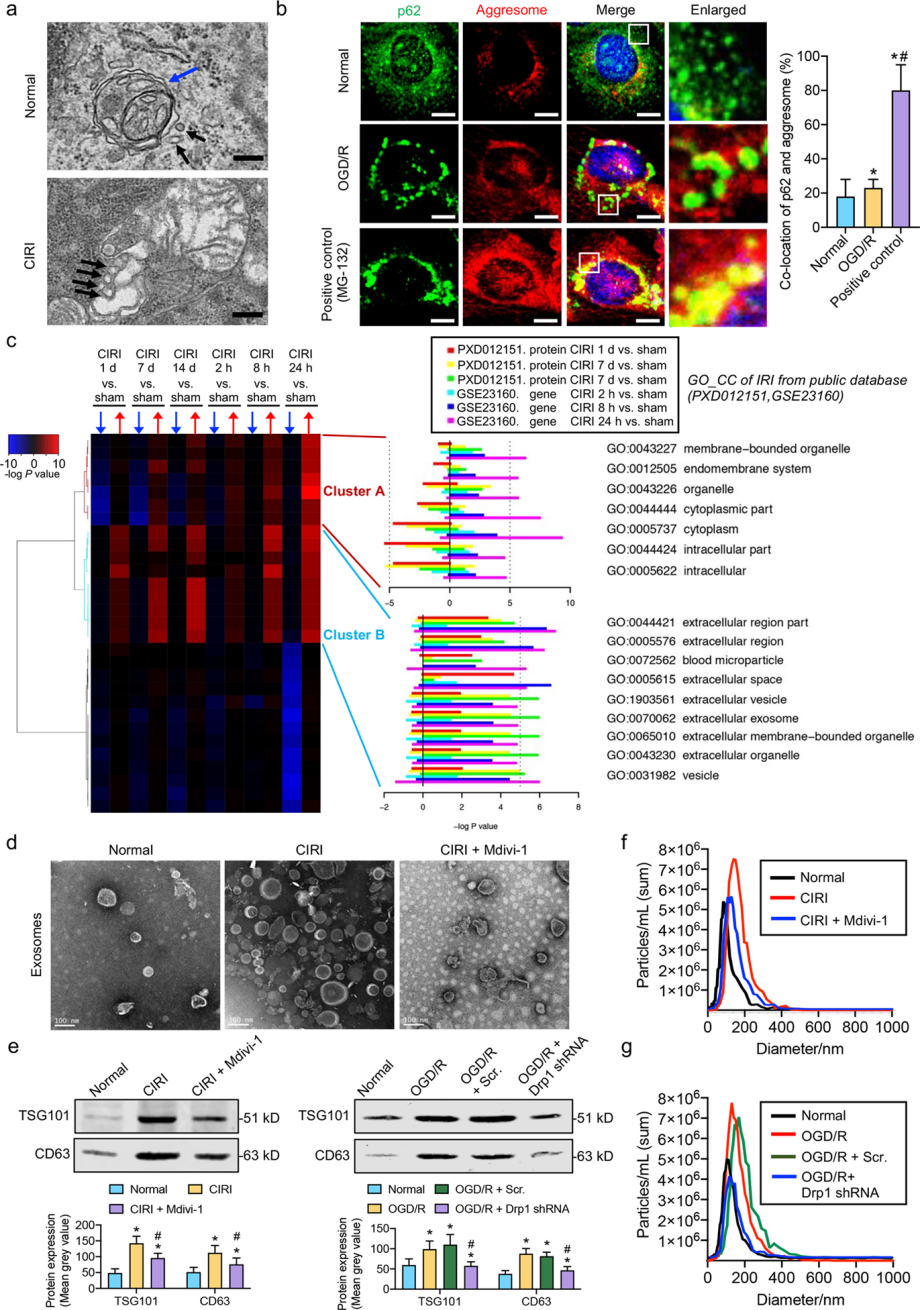
Undegraded p62-labeled autophagosomes induced by activated Drp1 are secreted via exosomes after CIRI and OGD/R. **a** TEM images showing autophagosome containing unhealthy mitochondria and membranous structure of suspected exosomes in the cerebral cortex after CIRI (bar = 200 nm). Blue arrow: autophagosome containing mitochondrial fragments. Black arrows: membranous structure of suspected exosomes. **b** Confocal images showing co-location of p62 and aggresomes in OGD/R-treated SH-SY5Y cells (bar = 10 μm). MG-132, a proteasome inhibitor, was used as a positive control (*n* = 5/group). **c** GO enrichment analysis on CIRI model sequencing data from the GEO database (NCBI ID: PXD012151 and GSE23160). **d** TEM images showing monolayer structures of exosomes after CIRI and Mdivi-1 treatment (bar = 100 nm). **e** Western blotting analysis showing the effects of Mdivi-1 or Drp1 shRNA on the expression of CD63 and TSG101 after CIRI or OGD/R (*n* = 8/group). **f** NTA particle analysis of exosomes in the cerebral cortex after CIRI and Mdivi-1 treatment. NTA ordinate refers to the number of exosomes, and the NTA abscissa refers to the diameters of the exosomes. **g** NTA particle analysis of exosomes in OGD/R-treated SH-SY5Y cells after Drp1 shRNA. NTA ordinate refers to the number of exosomes, and NTA abscissa refers to the diameters of the exosomes. ^*^P < 0.05 compared with normal group, ^#^*P* < 0.05 compared with CIRI or OGD/R group. CIRI cerebral ischemia–reperfusion injury, OGD/R oxygen–glucose deprivation/reoxygenation treatment, Scr. scramble-shRNA, TEM transmission electron microscope

Although our previous results have shown that activated Drp1-induced ROS accumulation may block autophagosome degradation via the RIP1/RIP3 pathway after CIRI, no large accumulation of autophagosomes was observed upon electron microscopy (Fig. 4a). This phenomenon was also confirmed in vitro as undegraded p62-labeled autophagosomes did not form aggresomes and accumulated in SH-SY5Y cells after OGD/R (Fig. 4b). The faith of the large numbers of undegraded p62-containing autophagosomes remains unknown.

We then performed GO enrichment analysis based on the public sequence database of the CIRI model (PXD012151 and GSE23160) and found that the membrane-bounded organelles were abnormally active after CIRI, which may be manifested in the release of large numbers of what seemed to be extracellular exosome or vesicle membranes (Fig. 4c). To verify the above bioinformatics analysis, we extracted exosomes from cerebral samples around CIRI infarcted area and detected a large number of monolayers with a diameter of 50–100 nm by electron microscopy (Fig. 4d), suggesting that large numbers of exosomes were secreted after CIRI. We further treated CIRI mice with Mdivi-1 and found that the number of exosomes was significantly decreased around the infarcted area (Fig. 4d), indicating that the increased exosome secretion may be related to Drp1 activation after CIRI. We then quantitatively detected the contents of exosomes with marker proteins CD63 and TSG101 by immunohistochemistry and Western blotting. The results showed that the increased CD63 and TSG101 expression after CIRI and OGD/R could be significantly inhibited by Mdivi-1 and Drp1 shRNA (Fig. 4e and Additional file 1: Fig. S4). The NTA particle analysis also confirmed the promoting effect of activated Drp1 on exosome secretion after CIRI (Fig. 4f; NTA ordinate, normal group: 5.6 × 10^6^ particles/ml; CIRI group: 7.5 × 10^6^ particles/ml; CIRI + Mdivi-1 group: 5.9 × 10^6^ particles/ml) and OGD/R (Fig. 4g; NTA ordinate, normal group: 5.1 × 10^6^ particles/ml; OGD/R group: 7.8 × 10^6^ particles/ml; OGD/R + Scr. group: 7.4 × 10^6^ particles/ml; OGD/R + Drp1 shRNA group: 4.1 × 10^6^ particles/ml). Besides, the NTA particle analysis further showed that the average diameter of exosomes (NTA abscissa) increased significantly after CIRI and OGD/R, which could be inhibited by Mdivi-1 or Drp1 shRNA (Fig. 4f, g). These results indicated that activated Drp1 increased the content enclosed in exosomes after CIRI and OGD/R. In summary, we speculated that the lack of large accumulations of p62-labeled autophagosomes after autophagic flux was blocked by the Drp1-ROS-RIP1/RIP3 axis may be due to the increased secretion of exosomes containing large numbers of undegraded autophagosomes after CIRI.

### Exosomes containing undegraded autophagosomes aggravate inflammation and further block autophagosome degradation via the RIP1/RIP3 pathway after CIRI

**Fig. 5:**
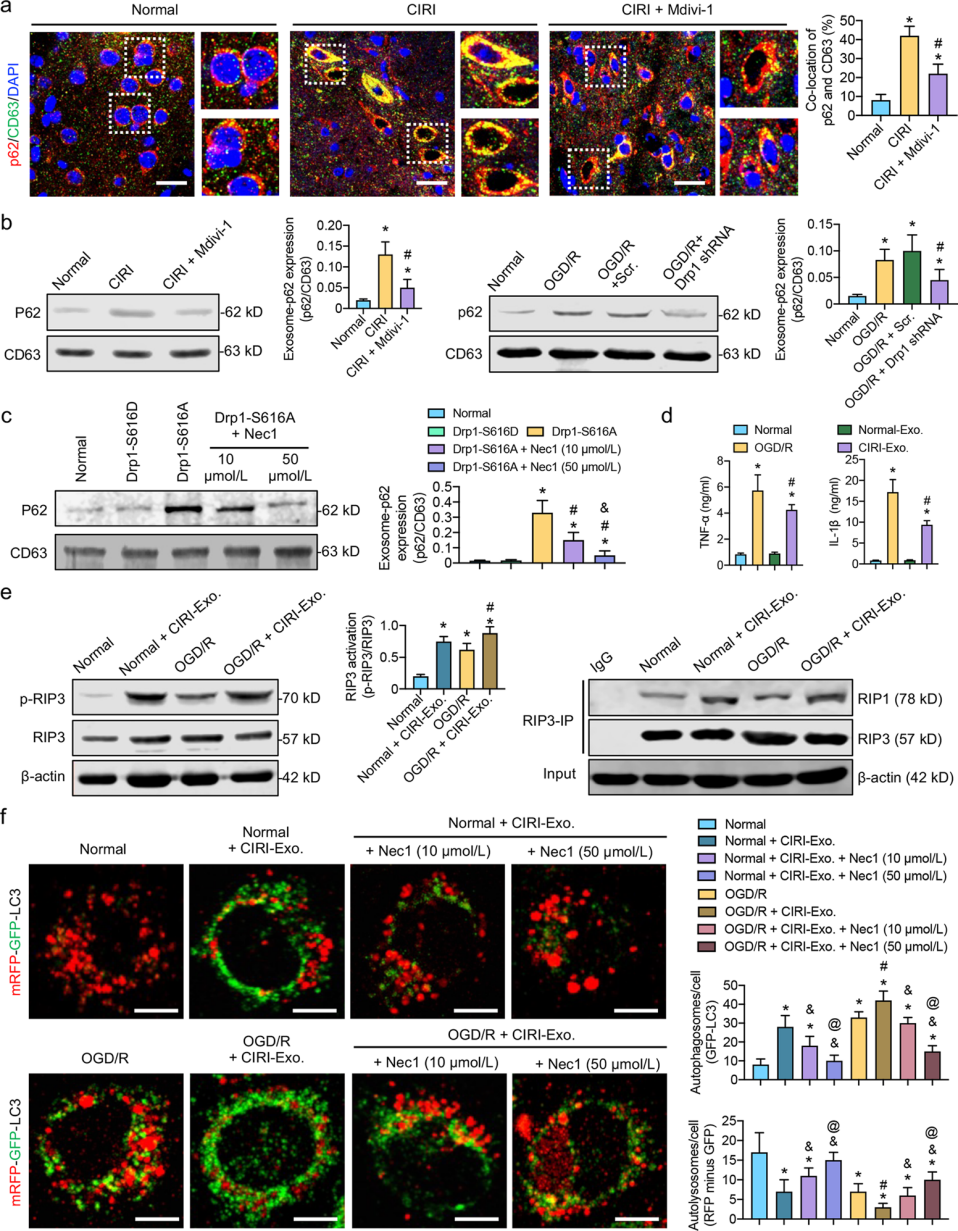
Exosomes containing p62-labeled autophagosomes aggravate inflammation and further stimulate the RIP1/RIP3 pathway to block autophagosome degradation after CIRI. **a** Immunofluorescence images showing the co-location of p62-labeld autophagosomes and CD63-labeled exosomes in the cerebral cortex after CIRI and Mdivi-1 treatment (bar = 20 μm, *n* = 5/group). **b** p62 expression in exosomes after CIRI or OGD/R and Mdivi-1 treatment or Drp1 shRNA (*n* = 5/group). **c** p62 expression in exosomes after Drp1 activation (S616A) and Nec1 treatment (10 μmol/L and 50 μmol/L, *n* = 5/group). **d** TNF-α and IL-1β levels in the culture supernatant of SH-SY5Y cells after being stimulated with normal or CIRI-derived exosomes (*n* = 5/group). **e** Western blotting analysis and Co-IP assay showing RIP3 phosphorylation and RIP1-RIP3 binding ability in SH-SY5Y cells after OGD/R and CIRI-derived exosomes stimulation (*n* = 5/group). **f** Immunofluorescence images showing autophagy flux labeled by mRFP-GFP-LC3 in SH-SY5Y cells after OGD/R, CIRI-derived exosomes stimulation, and Nec1 treatment (10 μmol/L and 50 μmol/L, bar = 10 μm, *n* = 5/group). ^*^P < 0.05, compared with normal group; ^#^*P* < 0.05, compared with CIRI or OGD/R group; ^&^*P* < 0.05, compared with CIRI-Exo. group; ^@^*P* < 0.05, compared with CIRI-Exo. + Nec1 (10 μmol/L) group. CIRI cerebral ischemia–reperfusion injury, Co-IP co-immunoprecipitation, Exo. exosomes, OGD/R oxygen–glucose deprivation/reoxygenation treatment

To verify our hypothesis, we observed the co-localization of CD63-labeled exosomes and p62-labeled autophagosomes in the cerebral cortex after CIRI. The immunofluorescence results showed that the co-localization of CD63 and p62 was significantly increased (*P* < 0.05, Fig. 5a and Additional file 1: Fig. S5), confirming that the secreted exosomes contained a large number of p62-mediated undegraded autophagosomes after CIRI. The co-localization of CD63 and p62 decreased by 47% after Drp1 activity was inhibited (*P* < 0.05, Fig. 5a). The Western blotting analysis further detected p62 content within the same number of exosomes and found that inhibiting Drp1 with Mdivi-1 or Drp1 shRNA may reduce the release of p62 autophagosome-containing exosomes after CIRI and OGD/R (Fig. 5b). We then activated Drp1 by the S616A mutation and extracted the same number of exosomes to detect the p62 content. The results showed that inhibiting the RIP1/RIP3 pathway dose-dependently reduced the p62 content in the exosomes after Drp1 activation (S616A) (Fig. 5c), suggesting that enhancing autophagosome degradation could reduce the release of exosomes containing p62 autophagosomes. Taken together, the above results confirmed that large numbers of undegraded p62-labeled autophagosomes were released in the form of exosomes after CIRI.

We then extracted exosomes from the cerebral cortex of normal and CIRI mice to stimulate normal SH-SY5Y cells for 24 h. Inflammatory factors, such as TNF-α and IL-1β, in the culture supernatant of the SH-SY5Y cells, were detected using ELISA kits. The results showed that the CIRI-derived exosomes, not normally-derived exosomes, could significantly increase TNF-α and IL-1β levels in normal SH-SY5Y cells, similar to OGD/R stimulation (Fig. 5d), indicating that exosomes containing undegraded autophagosomes may aggravate inflammation after CIRI.

Our results also showed that stimulating normal and OGD/R-treated SH-SY5Y cells with CIRI-derived exosomes could further activate the RIP1/RIP3 pathways to some extent (Fig. 5e). Meanwhile, the blocking effects of CIRI-mediated exosomes on autophagosome degradation may also be inhibited by Nec1, a RIP1/RIP3 inhibitor, in a dose-dependent manner (Fig. 5f). These results suggested that the inflammatory factors stimulated by exosomes after CIRI may further block autophagosome degradation via the RIP1/RIP3 pathway, triggering a pathophysiological vicious circle.

## Discussion

**Fig. 6:**
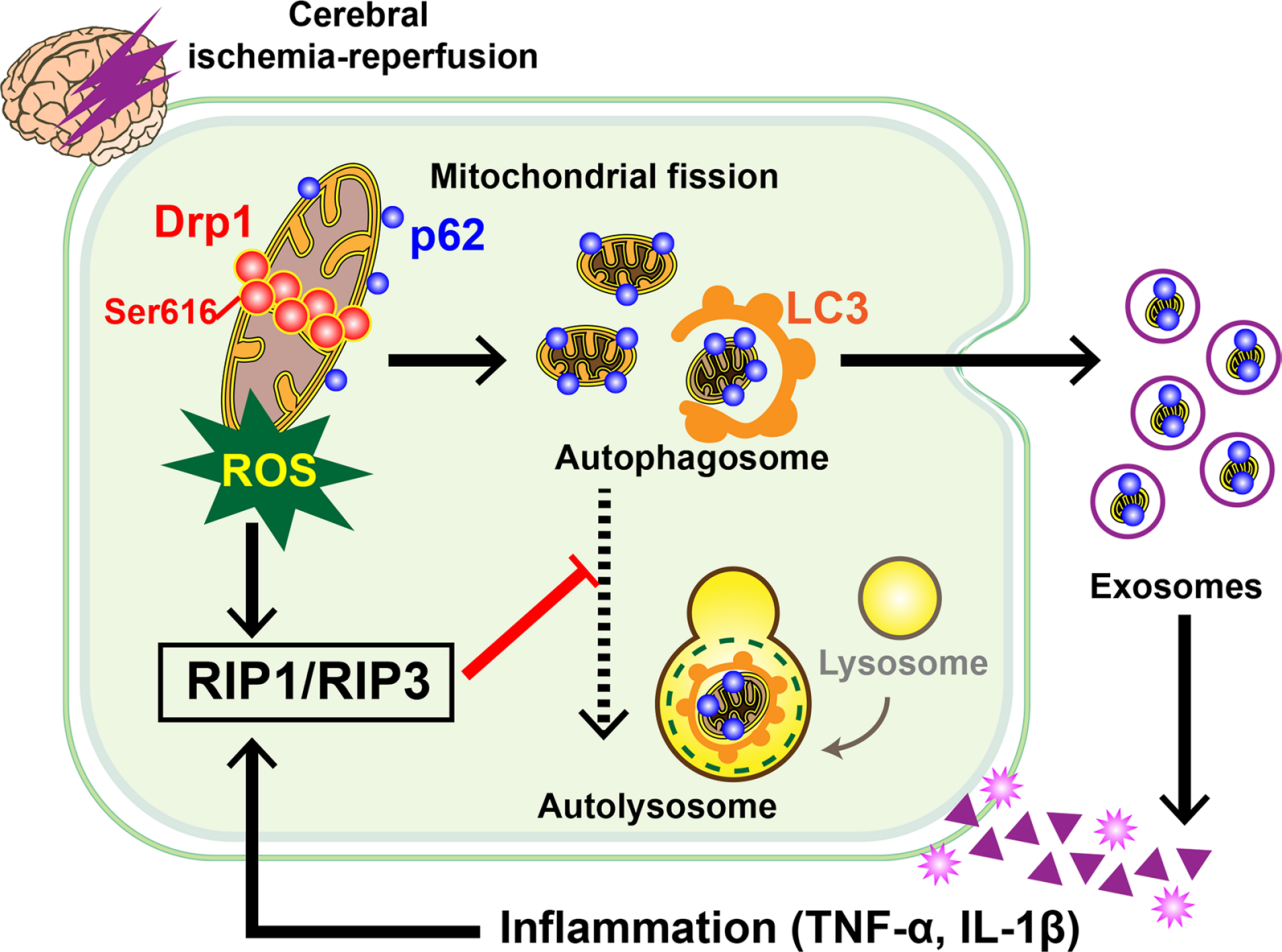
Scheme of activated Drp1 regulating p62-mediated autophagic flux and aggravating inflammation in CIRI. Drp1 activation following CIRI accelerates the p62-mediated formation of autophagosomes while inhibiting the autophagosome to autolysosome transition by activating the RIP1/RIP3 pathway. Undegraded autophagosomes are secreted extracellularly in the form of exosomes, causing an inflammatory response that further damages mitochondria, results in massive ROS generation, and blockage of autophagosomal degradation. CIRI cerebral ischemia–reperfusion injury, ROS reactive oxygen species

In this study, we found that activated Drp1 may accelerate p62-mediated autophagosome formation and block autophagosome degradation via the RIP1/RIP3 pathway following CIRI. The undegraded autophagosomes are secreted extracellularly as exosomes that cause inflammatory cascades, further damaging the mitochondria, leading to aggravated ROS accumulation, hindering autophagosome degradation, and ultimately establishing a pathophysiological vicious cycle (Fig. 6). Our study partly explains the mechanism underlying secondary injury caused by ROS and inflammatory cascades following CIRI, suggesting that measures aimed at mitochondrial protection and ROS scavenging may play a fundamental role in preventing and treating reperfusion injury. If Drp1-mediated mitochondrial homeostasis is not maintained and p62-mediated autophagic flux runs abnormally, the massive release of exosomes containing undegraded p62-labeled autophagosomes exacerbates the deterioration of ischemia-reperfusion through proinflammatory effects and oxidative stress after CIRI.

In recent years, mitochondrial quality control, especially mitochondrial fission, fusion, and autophagy, has received increasing attention in the occurrence, development, prevention, and treatment of acute ischemic and hypoxic injury diseases [19]. Under physiological conditions, the dynamic balance of mitochondrial fission and fusion maintains the normal mitochondrial morphology. In contrast, excessive mitochondrial fission and reduced mitochondrial fusion can lead to mitochondrial fragmentation and affect critical biological processes, such as mitochondrial DNA stability, energy synthesis, cell aging, and death. Autophagy plays an important role in mitochondrial homeostasis, ensuring that cells function normally via the timely removal of damaged or dysfunctional mitochondria. Cell damage occurs when mitophagy is impaired, and inadequate removal or excessive activation occurs [20]. In this study, we found a significant mitochondrial mass imbalance after CIRI, mainly manifested as an increased Drp1-mediated mitochondrial fission and a hampered p62-mediated autophagic flux. After CIRI, the phosphorylation level of Drp1-Ser616 in ischemic cerebral tissue was significantly higher, and a large amount of Drp1 was translocated from the cytoplasm to the mitochondria, unsetting the balance between mitochondrial fission and fusion, thereby resulting in morphological changes, such as mitochondrial fragmentation, and functional changes in mitochondrial ROS accumulation. Although the study of Calabrese et al. [21] mentioned that mitochondrial ROS may also aid recovery after injury, this mechanism was more likely involved with adaptive cellular responses under lower subtoxic levels of ROS after the chronic disease or preconditioning [22]. Severe acute injuries such as cerebral ischemia-reperfusion often caused excessive ROS accumulation, which could not be effectively cleared or transformed, thereby leading to decompensated cellular damage. Besides, Zhang et al. [23] demonstrated the nitrosylation of Drp1 in the cerebral tissue after CIRI. However, Lee et al. [24] concluded that Drp1 nitrosylation did not directly regulate Drp1 activity but instead led to excessive mitochondrial fission by upregulating Drp1-Ser616 phosphorylation. Therefore, we suggest that the increased phosphorylation of Drp1-Ser616 and its mitochondrial translocation underlies the mitochondrial mass imbalance that occurs after CIRI, and that this process can further affect the p62-mediated regulation of the autophagic flux.

Previous studies [8, 25, 26] on the regulation of the autophagic flux by mitochondrial fission reported that Drp1 could regulate autophagy through its interaction with a variety of LC3 adaptors and receptors. Wu et al. [27] showed that Drp1 could interact with the LC3 receptors FUNDC1 and BCL2L13 to induce autophagy; however, the specific regulatory mechanism was not fully elucidated. Our previous study [8] in vascular tissues from hemorrhagic shock mice found that Drp1 could inhibit the mitochondrial recruitment of Parkin by upregulating mitochondrial Clec16a to affect autophagy; however, the complete autophagic flux process was not explored in-depth. In the present study, we further investigated the regulatory effects of Drp1 on Parkin substrate protein p62 on the outer mitochondrial membrane, and found that activated Drp1 could accelerate the formation of p62-labeled autophagosomes, promoting p62 translocation from mitochondrial-bound to its free form. Yamada et al. [28] found a similar significant increase in mitochondrial p62 expression after Drp1 knockdown in nonalcoholic fatty liver. Thus, they proposed that p62 was more critical than Parkin in the process of autophagic flux [29]. Yamada et al. [30] also found an increase in mitochondrial p62 expression in the Purkinje neurons from Drp1 knockout mice and noted that further p62 knockdown could ameliorate the neuronal injury that results from Drp1 deficiency but could not change the effect of ROS accumulation on autophagic flux, which is consistent with our findings. The above studies suggest that Drp1 and p62 may antagonistically bind to the mitochondrial outer membrane. We hypothesize that this may be related to the competitive binding of Drp1 and p62 to a specific receptor protein on the mitochondrial outer membrane, whose underlying mechanism warrants further exploration.

However, reports that elevated p62 levels are accompanied by autophagy are not uncommon, which may be linked to the fact that related studies [31] have only examined the absolute expression of autophagy-related proteins without thoroughly examining the dynamic changes in autophagic flux. Studies have shown that the relationship between p62 and autophagy is bidirectional; on the one hand, intracellular p62 levels are strictly regulated by autophagic activity, and on the other hand, p62 can negatively regulate autophagic activity by activating the mTORC1 signaling pathway [32]. Given the complex relationship between p62 and autophagy, our study observed the whole process of autophagic flux, including autophagosome generation and autophagosome degradation. In addition to Drp1 activation accelerating the formation of p62-containing autophagosomes after CIRI, our study also found that Drp1-induced mitochondrial ROS accumulation could block autophagosome degradation via the RIP1/RIP3 pathway, leading to inefficient turnover of damaged mitochondria.

RIP1/RIP3 is a key pathway of programmed necrosis (necroptosis) ubiquitous in neurons. RIP1/RIP3 pathway reportedly promotes necroptosis by auto-phosphorylation under stimuli, such as TNF-α, thus playing a critical role in regulating inflammation in cerebral tissue [33]. Zhou et al. [34] showed that activated Drp1 could activate the NLRP3 inflammasome through the RIP1/RIP3 pathway in subarachnoid cavity hemorrhage. Rayamajhi et al. [35] showed that viral infection could activate Drp1 via the RIP1/RIP3 pathway, thereby damaging mitochondria and leading to ROS accumulation. The above studies suggest that there may be positive feedback regulation loops between Drp1 and the RIP1/RIP3 pathway, which are mutually causal; however, the specific mechanism remains to be elucidated. A recent study in intestinal epithelial cells [33] showed that the activation of the RIP1/RIP3 pathway significantly increased p62 co-localization with the autophagosome protein LC3, significantly reducing p62 co-localization with the autolysosome protein LAMP1, suggesting that the RIP1/RIP3 pathway might inhibit the conversion of autophagosome to autolysosome during autophagic flow, which is consistent with our findings. Moreover, Li et al. [36] showed that p62 could form a complex with RIP1/RIP3 and accelerate cardiomyocyte programmed necrosis after myocardial ischemia-reperfusion. This finding might also be related to our observation that the RIP1/RIP3 pathway inhibits p62-mediated autophagosome degradation, and we suggest that in-depth exploration of the autophagosome degradation mechanisms may have implications for the regulation of inflammation after acute ischemia-hypoxia injury.

Exosomes are monolayer vesicles with a diameter of 30–150 nm. The morphology, size, and contents of exosomes vary significantly under different external stimulation conditions [37]. Fan et al. [16] showed that intervention with Drp1 could not only improve mitochondrial function as well as mitophagy but also reduce the release and transmission of neuronal exosomes in Parkinson’s disease. Similarly, we also observed the promoting effect of activated Drp1 on the release of exosomes after CIRI, which could be markedly reduced after intervention with Drp1 in vivo and in vitro. Previous studies [38, 39] have shown that exosomes transmit information between cells by wrapping small molecules, such as mRNA, miRNA, and lipids. Recently, it has been found that exosomes can also play an important role in wrapping organelle fragments [40]. Islam et al. [41] showed that bone marrow mesenchymal stem cells could regulate alveolar surface activity and ATP content by releasing exosomes containing mitochondrial fragments into alveolar epithelial cells in acute lung injury. Phinney et al. [42] showed that mesenchymal stem cells could release exosomes containing depolarized mitochondria into the plasma membrane to regulate intracellular oxidative stress. At the same time, these exosomes could also be phagocytosed and recycled by macrophages to boost energy production. In this study, we found that undegraded p62-labeled autophagosomes are encapsulated in exosomes stimulated by activated Drp1 after CIRI. The inflammatory cascade triggered by this process can further activate the RIP1/RIP3 pathway, resulting in aggravated ROS accumulation and blockage of autophagosomal degradation, setting up a vicious cycle after CIRI. Our study further supplements the variety of exosome inclusions and confirms the bridging role of exosomes in regulating cell functions. Babuta et al. [43] also speculated that the increased release of exosomes might be related to impaired autophagic functions in alcoholic liver disease but did not elucidate the causal mechanism. By observing the relationship between intact autophagic flow and exosome release, our study has confirmed that the obstruction of autophagic flow leading to a large accumulation of undegraded p62-labeled autophagosomes might be the main cause for the increase of exosome release. Moreover, a large number of autophagy-related proteins, such as p62 and LC3, were also detected in a previous exosome MS/MS analysis [44], which is consistent with our results.

This study has a few limitations: (1) Our cell model used OGD/R-treated SH-SY5Y cells; whether our findings also apply to other cerebral tissue cell lines, such as BV2 and CIRI clinical samples, requires further validation. (2) In addition to the classical p62-mediated mitophagy pathway, whether other LC3-binding receptors, such as BNIP3, FUNDC1, and FKBP8, may also be involved in the Drp1-regulated autophagic flux after CIRI, warrants further investigation. (3) Due to the limitation of the current exosome detection methods without high temporal and spatial resolution-specific imaging, currently, we can only use exosome marker proteins combined with optical imaging technology for localization analysis. In the future, more accurate dynamic investigations need to be carried out at the single-cell and single-exosome levels. (4) In an actual scenario, cerebral ischemia-reperfusion usually occurs in cerebral blood flow blocked by clots and resumed by tissue plasminogen activator (t-PA). How would blood clots or t-PA affect the conclusion of this study need further investigation in clinical trials.

Nevertheless, this study suggests that accelerating p62 autophagosome conversion and clearing damaged mitochondria by regulating the RIP1/RIP3 pathway may be effective measures to ameliorate the pathophysiology of CIRI. Maintaining mitochondrial mass balance and reducing mitochondrial ROS accumulation (including reducing ROS generation and increasing ROS clearance) may be keys to fundamentally improving the prognosis of CIRI.

## Conclusions

In this study, we linked multiple pathophysiological processes, such as excessive mitochondrial fission, massive ROS accumulation, abnormal autophagic flux, increased exosome release, and inflammatory over-activation, which occur following CIRI and establish a vicious cycle that leads to cell death. Drp1 activation following CIRI accelerates the p62-mediated formation of autophagosomes while inhibiting the autophagosome to autolysosome transition by activating the RIP1/RIP3 pathway. Undegraded autophagosomes are secreted extracellularly in the form of exosomes, causing an inflammatory response that further damages mitochondria and results in massive ROS generation and the blockage of autophagosomal degradation. Our study systematically dissected the pathophysiological vicious cycle after CIRI from the perspective of mitochondrial damage, providing an experimental basis and intervention target for the prevention and treatment of CIRI.

## Supplementary Information

**Additional file** **1: Fig.S1. **Activationand distribution of Drp1 after CIRI and ODG/R. **Fig. S2. **Effects ofMdivi-1 on mitochondrial p62 release after CIRI. **Fig. S3. **Effects ofDrp1-induced ROS accumulation on RIP1/RIP3 pathway after cerebral I/R. **Fig.S4. **Effects of Mdivi-1 on CD63 expression after CIRI. **Fig. S5. **Effectsof Mdivi-1 on exosomes containing p62-labeled autophagosomes after CIRI.

## Data Availability

All data generated or analyzed during this study are included in this published article.

## Abbreviations

CIRI: Cerebral ischemia-reperfusion injury;
Co-IP: Co-immunoprecipitation
DCFH-DA: 2′,7′-Dichlorodihydrofluorescein diacetate
DHE: Dihydroethidium
Exo.: Exosomes
FBS: Fetal bovine serum
MCAO: Middle cerebral artery occlusion
mtDNA: Mitochondrial DNA
OGD/R: Oxygen–glucose deprivation/reoxygenation treatment
ROI: Region of interest
ROS: Reactive oxygen species
Scr.: Scramble-shRNA
TEM: Transmission electron microscope
t-PA: Tissue plasminogen activator
