# Supplementary material for: Activated Drp1 regulates p62-mediated autophagic flux and aggravates inflammation in cerebral ischemia-reperfusion via the ROS-RIP1/RIP3-exosome axis
Source: Mil Med Res. 2022 May 27;9:25. doi: 10.1186/s40779-022-00383-2 (PMC9137164; doi:10.1186/s40779-022-00383-2)
Supplement: Supplementary file 1 — Additional file 1: Fig.S1. Activationand distribution of Drp1 after CIRI and ODG/R. Fig. S2. Effects ofMdivi-1 on mitochondrial p62 release after CIRI. Fig. S3. Effects ofDrp1-induced ROS accumulation on RIP1/RIP3 pathway after cerebral I/R. Fig.S4. Effects of Mdivi-1 on CD63 expression after CIRI. Fig. S5. Effectsof Mdivi-1 on exosomes containing p62-labeled autophagosomes after CIRI. [file 40779_2022_383_MOESM1_ESM.pdf]

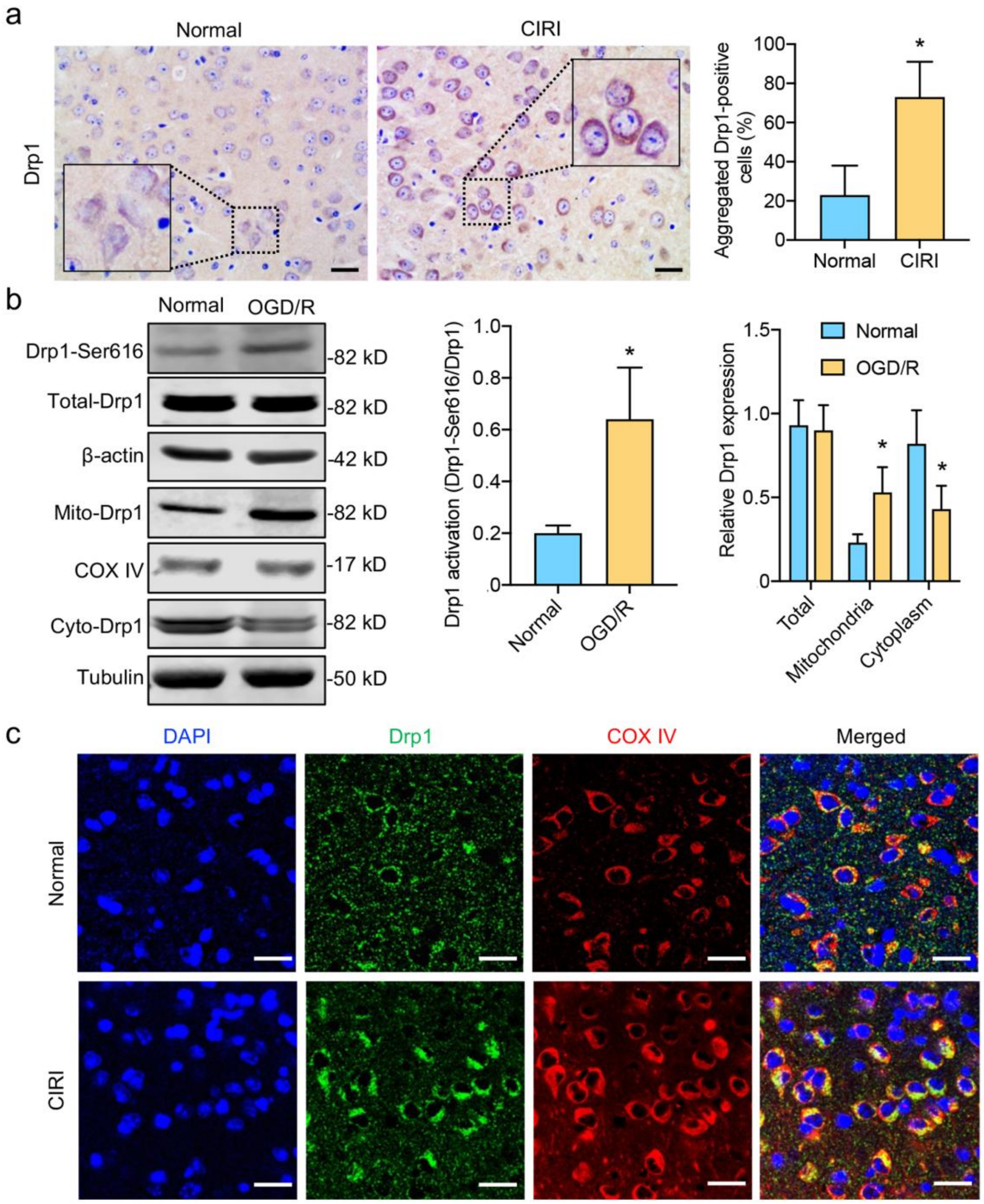

Fig. S1 Activation and distribution of Drp1 after CIRI and ODG/R. **a** Immunohistochemistry images showing Drp1 aggregation in the cerebral cortex after CIRI (bar = 25 μm). Quantitative data of aggregated Drp1-positive cells were calculated for each group. **b** Activation of Drp1-Ser616 and protein expression of Drp1 in total, cytoplasmic, and mitochondrial fractions after OGD/R. β-actin, COX IV, and tubulin were used as internal references for total, mitochondrial, and cytoplasmic fractions, respectively (*n* = 8/group). **c** Representative immunofluorescence images showing co-location of Drp1 and mitochondria (COX IV) in the cerebral cortex after CIRI (bar = 20 μm). Enlarged images are presented in Fig. 1f. $^{*}P < 0.05$ compared with the normal group. CIRI cerebral ischemia-reperfusion injury, OGD/R oxygen-glucose deprivation/reoxygenation treatment

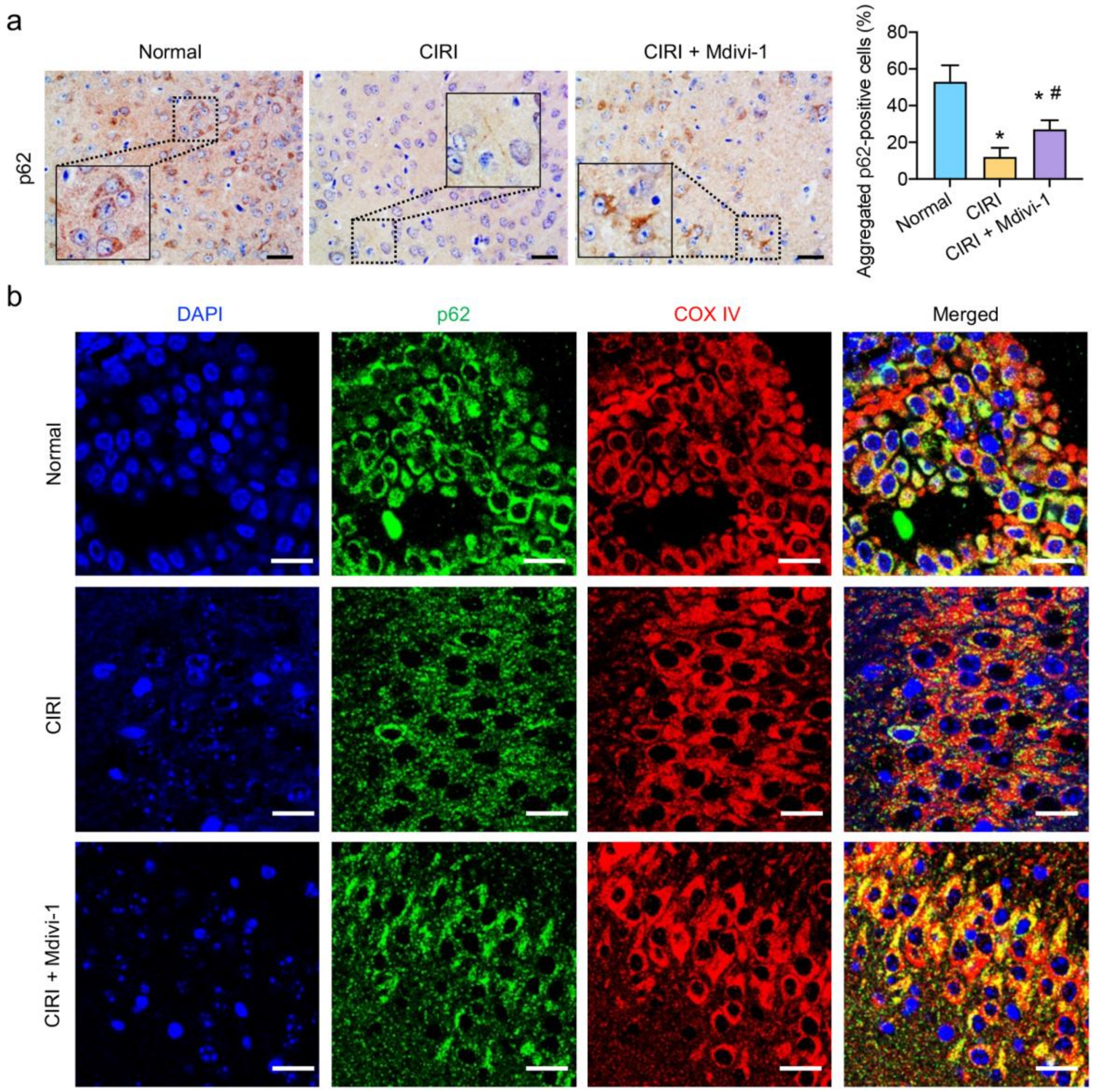

Fig. S2 Effects of Mdivi-1 on mitochondrial p62 release after CIRI. **a** Immunohistochemistry images showing p62 aggregation in the cerebral cortex after I/R and Mdivi-1 treatment (bar = 25 μm). Quantitative data of aggregated p62-positive cells were calculated for each group (*n* = 5/group). **b** Representative immunofluorescence images showing co-location of p62 and mitochondria (COX IV) in the cerebral cortex after CIRI and Mdivi-1 treatment (bar = 20 μm). These represent detailed images for Fig. 2a. $^{*}P < 0.05$ compared with normal group, $^{\#}P < 0.05$, compared with CIRI group. CIRI cerebral ischemia-reperfusion injury

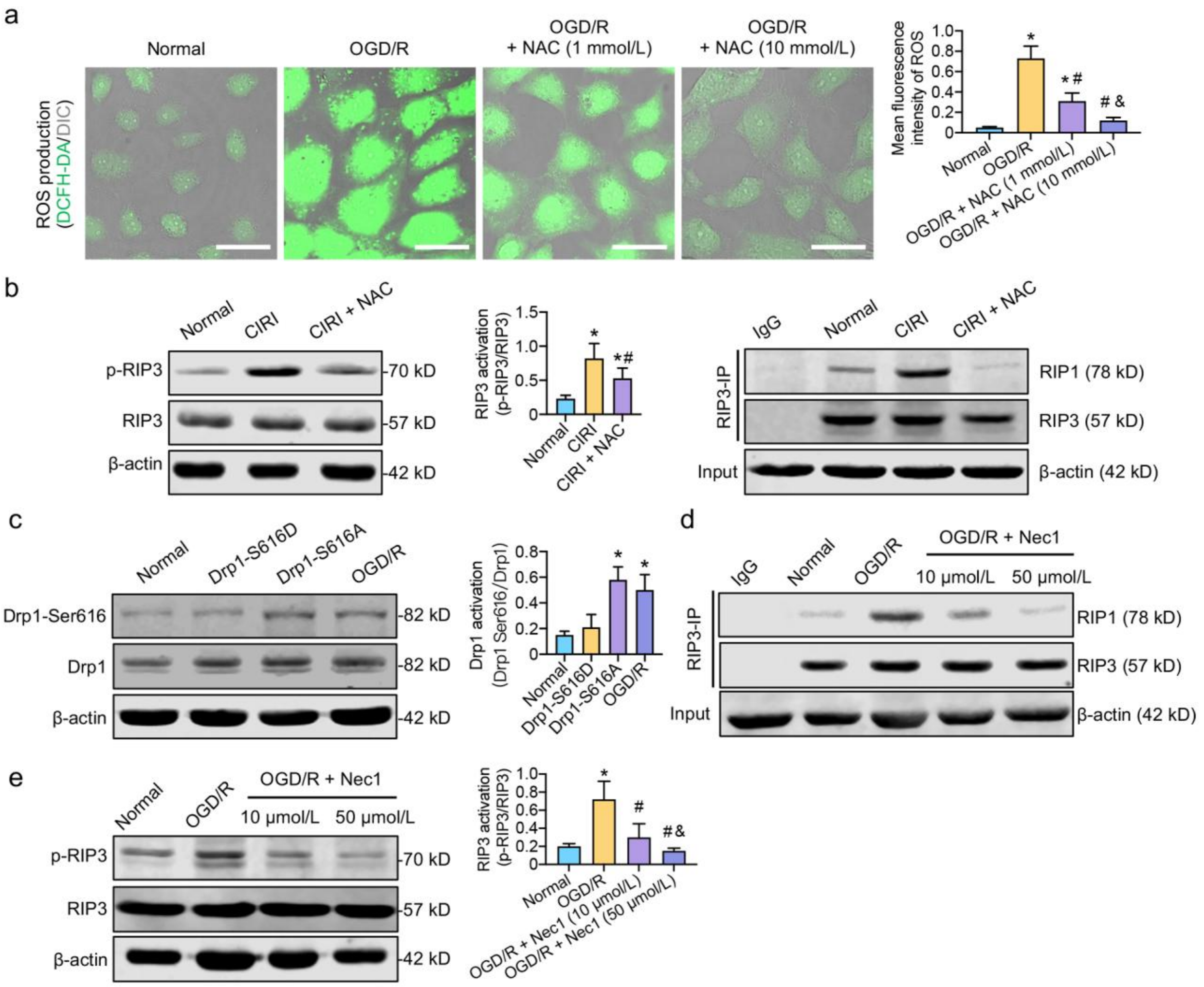

Fig. S3 Effects of Drp1-induced ROS accumulation on RIP1/RIP3 pathway after cerebral I/R. **a** Fluorescence intensity of ROS DCFH-DA showing the effects of NAC on ROS scavenging in OGD/R-treated SH-SY5Y cells (bar = 50 µm, *n* = 8/group). **b** Effects of NAC (25 mg/kg) on RIP3 phosphorylation and RIP1-RIP3 binding ability in the cerebral cortex after I/R (*n* = 8/group). **c** Validation experiment of Drp1 S616A mutation on Drp1 activation. OGD/R group was used as the positive control group (*n* = 8/group). **d** Validation experiment of Nec1 on inhibiting RIP1-RIP3 binding ability in OGD/R-treated SH-SY5Y cells. **e** Validation experiment of Nec1 on inhibiting RIP3 phosphorylation in OGD/R-treated SH-SY5Y cells (*n* = 8/group). $^{*}P < 0.05$ compared with normal group, $^{\#}P < 0.05$ compared with IR or OGD/R group, $^{\&}P < 0.05$ compared with OGD/R + NAC (1 mmol/L) or OGD/R + Nec1 (10 µmol/L) group. DCFH-DA 2',7'-dichlorodihydrofluorescein diacetate, OGD/R oxygen-glucose deprivation/reoxygenation treatment, ROS reactive oxygen species

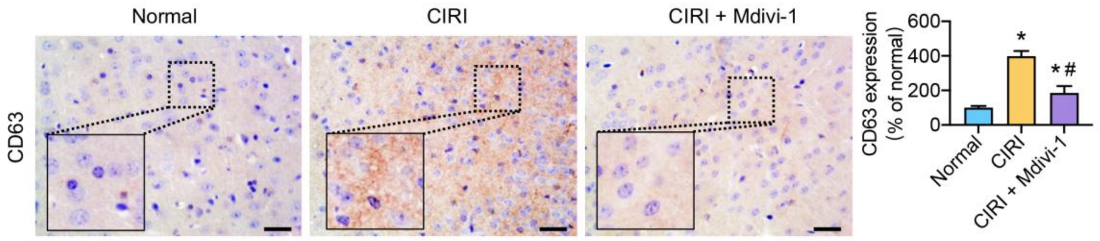

Fig. S4 Effects of Mdivi-1 on CD63 expression after CIRI. Immunohistochemistry images showing CD63 expression in the cerebral cortex after CIRI and Mdivi-1 treatment (bar = 25 μm; n = 5/group). $^{*}P < 0.05$, compared with normal group; $^{\#}P < 0.05$, compared with CIRI group. CIRI cerebral ischemia-reperfusion injury

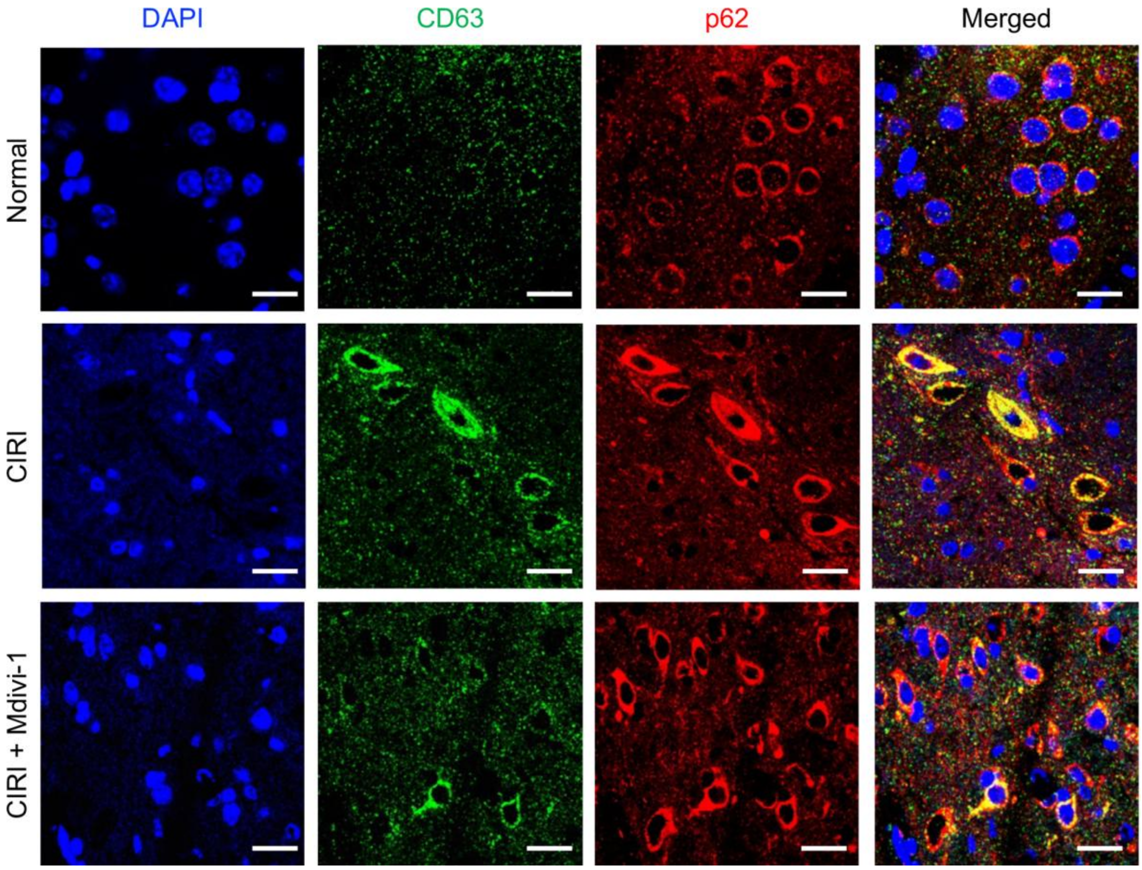

Fig. S5 Effects of Mdivi-1 on exosomes containing p62-labeled autophagosomes after CIRI. Representative immunofluorescence images showing the co-location of p62-labeld autophagosomes and CD63-labeled exosomes in the cerebral cortex after CIRI and Mdivi-1 treatment (bar = 20 μm). These are detailed images for Fig. 5a. CIRI cerebral ischemia- reperfusion injury
